# Using read codes to identify patients with irritable bowel syndrome in general practice: a database study

**DOI:** 10.1186/1471-2296-14-183

**Published:** 2013-12-02

**Authors:** Elaine F Harkness, Laura Grant, Sarah J O’Brien, Carolyn A Chew-Graham, David G Thompson

**Affiliations:** 1 Institute of Inflammation and Repair, Stopford Building, University of Manchester, Oxford Road, Manchester M13 9PL, UK; 2 Institute of Infection and Global Health, Leahurst Campus, University of Liverpool, Chester High Road, Neston, South Wirral CH64 7TE, UK; 3 Research Institute, Primary Care and Health Sciences, Keele University, Keele, Staffordshire ST5 5BG, UK; 4 Gastrointestinal Centre, Institute of Inflammation & Repair, University of Manchester, Clinical Sciences Building, Salford Royal NHS Foundation Trust, Stott Lane, Salford M6 8HD, UK

**Keywords:** Irritable bowel syndrome, Read Codes, Functional gastrointestinal disorders, Medically unexplained symptoms, Primary care, General practitioners

## Abstract

**Background:**

Estimates of the prevalence of irritable bowel syndrome (IBS) vary widely, and a large proportion of patients report having consulted their general practitioner (GP). In patients with new onset gastrointestinal symptoms in primary care it might be possible to predict those at risk of persistent symptoms. However, one of the difficulties is identifying patients within primary care. GPs use a variety of Read Codes to describe patients presenting with IBS. Furthermore, in a qualitative study, exploring GPs’ attitudes and approaches to defining patients with IBS, GPs appeared reluctant to add the IBS Read Code to the patient record until more serious conditions were ruled out. Consequently, symptom codes such as 'abdominal pain’, 'diarrhoea’ or 'constipation’ are used. The aim of the current study was to investigate the prevalence of recorded consultations for IBS and to explore the symptom profile of patients with IBS using data from the Salford Integrated Record (SIR).

**Methods:**

This was a database study using the SIR, a local patient sharing record system integrating primary, community and secondary care information. Records were obtained for a cohort of patients with gastrointestinal disorders from January 2002 to December 2011. Prevalence rates, symptom recording, medication prescribing and referral patterns were compared for three patient groups (IBS, abdominal pain (AP) and Inflammatory Bowel Disease (IBD)).

**Results:**

The prevalence of IBS (age standardised rate: 616 per year per 100,000 population) was much lower than expected compared with that reported in the literature. The majority of patients (69%) had no gastrointestinal symptoms recorded in the year prior to their IBS. However a proportion of these (22%) were likely to have been prescribed NICE guideline recommended medications for IBS in that year. The findings for AP and IBD were similar.

**Conclusions:**

Using Read Codes to identify patients with IBS may lead to a large underestimate of the community prevalence. The IBS diagnostic Read Code was rarely applied in practice. There are similarities with many other medically unexplained symptoms which are typically difficult to diagnose in clinical practice.

## Background

Irritable Bowel Syndrome (IBS) is a common gastrointestinal condition defined as 'a functional bowel disorder in which abdominal pain or discomfort is associated with defecation or a change in bowel habit, and with features of disordered defecation’ [1]. Prevalence estimates for IBS appear to vary widely according to the criteria used, population studied, the mode of study delivery and type of prevalence estimate. Typically estimates range between 2% and 22% in western countries [2]. In the UK, a community survey estimated the prevalence of IBS to be 10.5% with over half of patients having consulted their general practitioner (GP) within the past six months [3].

Medical management of IBS symptoms is empiric usually involving anti-spasmodics, anti-diarrhoeals or laxatives as appropriate to symptoms; and antidepressants, particularly low-dose tricyclic antidepressants [4]. Unfortunately, for some patients medical management is unsatisfactory. For patients with symptoms resistant to conventional medical therapy, current guidelines recommend referral for psychological intervention to cognitive behavioural therapy (CBT) or hypnotherapy [4].

Halder et al. [5] showed that in patients with new onset gastrointestinal symptoms in primary care it might be possible to predict those at risk of persistent symptoms. Thus it might be possible to identify those patients at risk of doing worse and fast-track them to these therapies. This should lead to less distress and lower healthcare utilisation for these patients in the long run. We aimed to test this finding prospectively as a part of a NIHR (National Institute for Health Research) programme of work (RP-PG-0407-10136). However, one of the difficulties encountered in previous work on IBS within primary care is the identification of patients.

GPs use a variety of Read Codes to describe patients presenting with IBS. Furthermore, in a qualitative study to explore GPs attitudes and approaches to defining, diagnosing and managing patients with IBS in primary care we found that, despite recent guidelines from the National Institute for Health and Clinical Excellence (NICE) [4], IBS is still regarded as a diagnosis of exclusion, and GPs are reluctant to add the IBS Read Code to the patient record until more serious conditions are ruled out [6]. As a consequence, symptom codes such as 'abdominal pain’ , 'diarrhoea’ or 'constipation’ are Read coded in the patient record. To our knowledge the range of Read Codes used by GPs to define IBS has not previously been investigated. We therefore used a database study to investigate the use of Read Codes in patients with IBS. In addition, two recent systematic reviews have found that the quality of coding of morbidities within primary care varied, and this has been attributed to the distinctiveness of the diagnosis [7,8]. We therefore also wished to explore the differences in coding between a 'functional’ and an 'organic’ disorder.

### Aim

The aim of the current study was to investigate the prevalence of recorded consultations for IBS and to explore the symptom profile of patients with IBS using data from the Salford Integrated Record (SIR). Patients with IBS were our primary group of interest, but we were also interested in how GPs coded patients with other gastrointestinal conditions, to determine whether there were any differences in coding practices across conditions. Patients with abdominal pain (AP) were chosen because it is the main feature of IBS, and must be present with two other symptoms to fulfil a diagnosis of IBS [4]. In addition, we selected patients with IBD because IBD is an organic disorder in which patients present with similar symptoms to IBS and we wished to explore differences in coding practices between a 'functional’ and an 'organic’ disorder.

## Methods

### Setting

The setting for this study was Salford Primary Care Organisation, North West of England with an estimated population of 228,992 in 2010 [9].

### Population

All patients registered with the 52 General Practices in Salford Primary Care Organisation.

### Data collection and coding

The SIR is a local patient sharing record system which integrates primary care, community care and secondary care information into one continuous electronic health record per patient. It is especially important for patients with long-term conditions, who may see many health professionals, saving the patient from having to repeat the same information multiple times. An anonymised dataset is made available for research through the auspices of North West e-Health.

Electronic clinical records from the SIR were obtained for a cohort of patients based on symptom and diagnostic codes for gastrointestinal disorders for the period January 2002 to December 2011. Information was supplied in two separate files: the first contained anonymised patient identifiers, sex and year of birth; the second contained journal entry identifier, anonymised patient identifier, date of journal entry, Read Code description and Read Code. The files were matched on patient identifier, and checked for duplicates and anomalies. Duplicate records and patients under the age of 17 were removed. Read Codes were then coded into a new variable to distinguish between symptom and/or diagnostic codes, medication codes and referral codes.

### Analysis

For the study we carried out analysis on three groups. These were patients with:

a) IBS (Read Codes 14CF. and J521. including any subheadings)

b) abdominal pain (Read Codes 196.., 197.. and R090. including any subheadings)

c) Inflammatory Bowel Disease (IBD) (Read Codes J4… (without subheadings), J40.. and J41.. including any subheadings)

For each group of patients we identified an index episode of IBS, abdominal pain or IBD. For each index episode, an index date was created based on the date for that particular journal entry. For each condition we then looked one year pre and post the index date to determine:

i) Symptoms/diagnoses recorded pre and post IBS, abdominal pain and IBD

ii) Medications prescribed pre and post IBS, abdominal pain and IBD

iii) Referrals to gastrointestinal (GI) specialists pre and post IBS, abdominal pain and IBD

The index episode was taken as the first occurrence of IBS, abdominal pain or IBD. The date of this episode was then used to calculate the number of days between the index episode and all corresponding journal entries for the same patient. Where the journal entry date was the same as the index date symptoms/diagnoses were included in the previous year and medications and referrals were included in the year after the index episode. Pre- episode data was based on those with an index episode between 2003 and 2011 (so that only those with a complete year of data available before the index year were included), likewise post-episode data was based on those with an index episode between 2002 and 2010.

We also calculated prevalence estimates based on Salford population data. Population data was obtained from the Office of National Statistics (ONS) website for Salford Primary Care Organisation [9]. Rates were standardised to the Greater Manchester population for 2006. This includes the population of the ten primary care organisations within the Greater Manchester area, which is roughly 2.5 million.

## Results

There were 8,444 patients in Salford with an IBS Read Code recorded in the years 2002 to 2011. AP and IBD were Read Coded for 42,490 and 1,510 patients respectively.

IBS and AP were much more common in females, whereas a similar proportion of males and females had IBD (Table 1). The highest proportion of those with IBS, AP or IBD was in those aged 18–39 and declined with age. More patients with IBD were aged 60 or over (26%) compared with those aged 60 or over with IBS (16%) and AP (22%).

**Table 1:** Demographic information for patients with IBS, AP and IBD, Salford 2002-2011

		**IBS**		**AP**		**IBD**	
		**n**	**%**	**n**	**%**	**n**	**%**
Female		6137	72.7	26071	61.4	755	50.0
Age	18-39	4373	51.8	19662	46.3	629	41.7
	40-59	2703	32.0	13379	31.5	487	32.3
	60+	1368	16.2	9449	22.2	394	26.1

### Prevalence estimates

**Figure 1:**
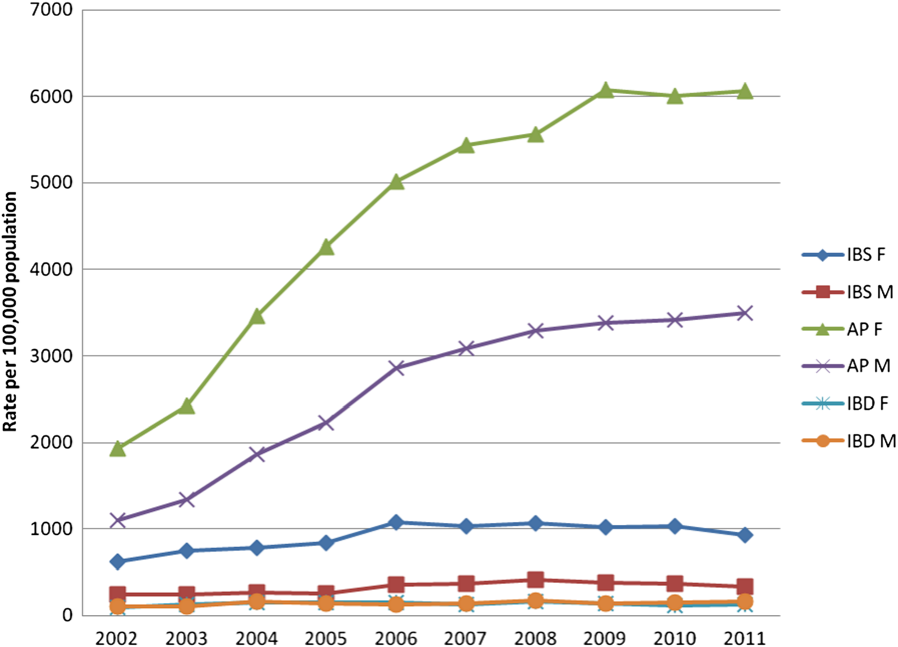
Age standardised rates, Salford 2002–2011.

The age standardised rates for IBS, AP and IBD per year per 100,000 population were 616, 3,606 and 139 respectively. Figure 1 shows the age standardised rates per 100,000 population for IBS, AP and IBD by gender in Salford for the years 2002–2011. Rates have been standardised to the Greater Manchester population. The rates for IBD have remained stable across the ten year period for both males and females. IBS shows a similar pattern in males and females with a slight increase in prevalence rates up to 2006 before levelling out over the second half of the ten year period. The rates of AP on the other hand increased considerably in both males and females from 2002 to about 2009, after which they have levelled out for both males and females.

### Symptoms pre and post IBS, AP or IBD

Table 2 shows the number of symptoms/diagnoses one year prior to and one year after the index episode of IBS, AP and IBD. The most commonly reported symptoms in the year prior to IBS were AP (19.5%), diarrhoea (6.1%) and bloating (3.8%). However, for most patients (69.4%) no GI symptoms were recorded in the year prior to their IBS. Likewise in the year after the index episode of IBS most patients did not have any GI symptoms recorded (79.1%), AP was the most commonly recorded symptom (13.4%) and 14% of patients had a further episode of IBS recorded in the year after their index episode. IBD showed a similar pattern to IBS with the majority of patients (69.6%) not having any symptoms recorded in the year prior to their IBD diagnosis. The most commonly reported symptoms were AP (15.9%) and diarrhoea (11.8%). In the year after the index episode of IBD, AP and diarrhoea were still the most commonly reported symptoms but the proportions tended to be lower (10.4% and 5.4% respectively). Almost a third of patients had a further episode of IBD recorded in the year after their index episode.

**Table 2:** Number (%) of symptoms pre and post IBS, AP and IBD, Salford 2002-2011

	**Pre IBS (n = 7728)**		**Post IBS (n = 7665)**		**Pre AP (n = 39974)**		**Post AP (n = 37851)**		**Pre IBD (n = 1336)**		**Post IBD (n = 1382)**	
Symptoms/diagnoses*	**n**	**%**	**n**	**%**	**n**	**%**	**n**	**%**	**n**	**%**	**n**	**%**
Abdominal pain	1509	19.5	1028	13.4	-	-	8683	22.9	212	15.9	144	10.4
Bloating	291	3.8	137	1.8	411	1.0	374	1.0	8	0.6	7	0.5
Constipation symptom	166	2.1	138	1.8	639	1.6	700	1.8	16	1.2	14	1.0
Change in bowel habit	130	1.7	45	0.6	175	0.4	174	0.5	46	3.4	5	0.4
Diarrhoea symptoms	475	6.1	256	3.3	1046	2.6	972	2.6	158	11.8	74	5.4
Nausea	104	1.3	100	1.3	483	1.2	513	1.4	6	0.4	12	0.9
Vomiting	72	0.9	67	0.9	583	1.5	583	1.5	11	0.8	19	1.4
Diarrhoea & vomiting	47	0.6	43	0.6	202	0.5	192	0.5	12	0.9	6	0.4
Tenesmus	2	0.0	2	0.0	6	0.0	12	0.0	2	0.1	0	0.0
Faeces/motion symptoms	31	0.4	34	0.4	72	0.2	80	0.2	8	0.6	4	0.3
None of the above symptoms	5367	69.4	6062	79.1	36994	92.5	27010	71.4	930	69.6	1143	82.7
1 of the above symptoms	1950	25.2	1380	18.0	2699	6.8	9555	25.2	339	25.4	200	14.5
2 or more of the above symptoms	411	5.3	223	2.9	281	0.7	1286	3.4	67	5.0	39	2.8
Functional constipation	151	2.0	121	1.6	511	1.3	573	1.5	20	1.5	21	1.5
Functional diarrhoea	31	0.4	31	0.4	75	0.2	118	0.3	22	1.6	15	1.1
GI infection	84	1.1	63	0.8	295	0.7	295	0.8	18	1.3	8	0.6
IBS	-	-	1090	14.2	747	1.9	1061	2.8	42	3.1	25	1.8
IBD	33	0.4	40	0.5	128	0.3	204	0.5	-	-	391	28.3

*Symptoms on the same day were coded as pre.

For patients with AP most patients (92.5%) had no symptoms recorded in the year prior to their index episode of AP. In the year after their index episode, 23% of patients had a further AP episode, and 2.8% of patients had an IBS diagnosis.

The proportion of patients who reported two or more symptoms in the previous year or the year after IBS, AP or IBD was coded in 5% or less in most instances.

### Medications pre and post IBS, AP or IBD

Table 3 shows the number of NICE guideline recommended medications for IBS prescribed in the year prior to and after the index episode of IBS, AP and IBD. NICE guideline recommended medications for IBS were prescribed for 31% and 54% of patients in the year prior to and after their index episode of IBS respectively. The increased use of these medications was most marked for antispasmodics which increased from 17% in the year prior to IBS to 44% in the year after. IBS NICE guideline recommended medications were also commonly prescribed in patients with AP and IBD, with about a third of patients being prescribed medications in the year after the index episode of AP or IBD. Prescribing of antispasmodics increased markedly from 5% to 19% in those with AP pre- and post- the index episode. Approximately 10% of patients with IBS, AP or IBD were prescribed selective serotonin reuptake inhibitors (SSRIs), and about 5% tricyclic antidepressants.

**Table 3:** Number (%) of medications pre and post IBS, AP and IBD, Salford 2002-2011

	**Pre IBS (n = 7728)**		**Post IBS (n = 7665)**		**Pre AP (n = 39974)**		**Post AP (n = 37851)**		**Pre IBD (n = 1336)**		**Post IBD (n = 1382)**	
**Medications**^**†**^	**n**	**%**	**n**	**%**	**n**	**%**	**n**	**%**	**n**	**%**	**n**	**%**
NICE medications	2395	31.0	4099	53.5	8560	21.4	13871	36.6	389	29.1	442	32.0
Bulking laxatives	288	3.7	629	8.2	754	1.9	1561	4.1	40	3.0	50	3.6
Stimulant laxatives	204	2.6	247	3.2	1106	2.8	1688	4.5	36	2.7	59	4.3
Faecal softeners	0	0.0	0	0.0	0	0.0	0	0.0	0	0.0	0	0.0
Osmotic laxatives	216	2.8	289	3.8	845	2.1	1550	4.1	27	2.0	50	3.6
Antimotility drugs	246	3.2	429	5.6	727	1.8	979	2.6	132	9.9	169	12.2
Antispasmodic drugs	1328	17.2	3397	44.3	1890	4.7	7035	18.6	157	11.8	149	10.8
Tricyclic antidepressants	397	5.1	553	7.2	1924	4.8	2221	5.9	66	4.9	78	5.6
SSRI	808	10.5	957	12.5	3530	8.8	3955	10.4	101	7.6	129	9.3
Other antidepressants	124	1.6	147	1.9	554	1.4	660	1.7	12	0.9	15	1.1

^†^Medications on the same day were coded as post.

Tables 4 and 5 show the number of NICE guideline recommended medications prescribed in the year prior to and year after the index episodes of IBS, AP and IBD for those with and without symptoms respectively. Where GI symptoms were recorded in the year prior to or the year after the index episode, the proportion of patients prescribed NICE guideline recommended medications was higher than in those without symptoms. However, a number of patients still received NICE guideline recommended medications in the year prior to their index episode of IBS (22%), AP (18%) or IBD (20%) despite not having any symptoms recorded in the year prior to their index episode. In those with IBS approximately 50% of those with no symptoms recorded in the year after their index episode were prescribed NICE guideline recommended medications, compared with about 30% in those with AP or IBD. In those where symptoms had been recorded in the year prior to or after the index episode the proportion prescribed NICE guideline recommended medications was much higher compared to those where no symptoms had been recorded.

**Table 4:** Number (%) of medications pre and post IBS, AP, IBD in those with no symptoms, Salford 2002-2011

	**Pre IBS (n = 5367)**		**Post IBS (n = 6062)**		**Pre AP (n = 36670)**		**Post AP (n = 27010)**		**Pre IBD (n = 930)**		**Post IBD (n = 1143)**	
**Medications**^**†**^	**n**	**%**	**n**	**%**	**n**	**%**	**n**	**%**	**n**	**%**	**n**	**%**
NICE medications	1176	21.9	2974	49.1	6720	18.3	8018	29.7	191	19.9	300	26.2
Bulking laxatives	119	2.2	437	7.2	569	1.6	840	3.1	21	2.2	32	2.8
Stimulant laxatives	77	1.4	129	2.1	795	2.2	896	3.3	19	2.0	33	2.9
Faecal softeners	0	0.0	0	0.0	0	0.0	0	0.0	0	0.0	0	0.0
Osmotic laxatives^‡^	93	1.7	152	2.5	571	1.6	745	2.8	13	1.4	25	2.2
Antimotility drugs	109	2.0	284	4.7	423	1.2	450	1.7	66	6.9	119	10.4
Antispasmodic drugs	533	9.9	2430	40.1	1414	3.9	3870	14.3	55	5.7	85	7.4
Tricyclic antidepressants	228	4.2	372	6.1	1647	4.5	1364	5.0	46	4.8	52	4.5
SSRI	471	8.8	680	11.2	3061	8.3	2488	9.2	59	6.1	92	8.0
Other antidepressants	55	1.0	84	1.4	472	1.3	383	1.4	8	0.8	10	0.9

^†^Medications on the same day were coded as post.

^‡^Excludes lactulose which is not recommended in NICE guidelines.

**Table 5:** Number (%) of medications pre and post IBS, AP, IBD in those with symptoms, Salford 2002-2011

	**Pre IBS (n = 2361)**		**Post IBS (n = 1603)**		**Pre AP (n = 3304)**		**Post AP (n = 10841)**		**Pre IBD (n = 406)**		**Post IBD (n = 239)**	
**Medications**^**†**^	**n**	**%**	**n**	**%**	**n**	**%**	**n**	**%**	**n**	**%**	**n**	**%**
NICE medications	1181	50.0	1103	68.8	1466	44.4	5314	49.0	189	46.6	135	56.5
Bulking laxatives	169	7.2	192	12.0	185	5.6	721	6.7	19	4.7	18	7.5
Stimulant laxatives	127	5.4	118	7.4	311	9.4	792	7.3	17	4.2	26	10.9
Faecal softeners	0	0.0	0	0.0	0	0.0	0	0.0	0	0.0	0	0.0
Osmotic laxatives^‡^	123	5.2	137	8.5	274	8.3	805	7.4	14	3.4	25	10.5
Antimotility drugs	137	5.8	145	9.0	304	9.2	529	4.9	66	16.3	50	20.9
Antispasmodic drugs	795	33.7	967	60.3	476	14.4	3165	29.2	102	25.1	64	26.8
Tricyclic antidepressants	169	7.2	181	11.3	277	8.4	857	7.9	20	4.9	26	10.9
SSRI	337	14.3	277	17.3	469	14.2	1467	13.5	42	10.3	37	15.5
Other antidepressants	69	2.9	63	3.9	82	2.5	277	2.6	4	1.0	5	2.1

^†^Medications on the same day were coded as post.

^‡^Excludes lactulose which is not recommended in NICE guidelines.

### Referrals pre and post IBS, AP or IBD

About 4% of patients had a gastrointestinal secondary care referral either in the year prior to or the year after their index episode of IBS (Table 6). This was in contrast to patients with IBD where 9% of patients had a GI referral in the year prior to their index episode and 22% had a GI referral in the year after their index episode. Few patients (0.9%) with AP had a GI referral in the year prior to their index episode. The number of referrals for mental health or lifestyle was low (0.0% to 0.7%) for all three conditions.

**Table 6:** Number (%) of referrals pre and post IBS, AP and IBD, Salford 2002–2011

	**Pre IBS (n = 7728)**		**Post IBS (n = 7665)**		**Pre AP (n = 39974)**		**Post AP (n = 37851)**		**Pre IBD (n = 1336)**		**Post IBD (n = 1382)**	
**Referrals**^**#**^	**n**	**%**	**n**	**%**	**n**	**%**	**n**	**%**	**n**	**%**	**n**	**%**
Gastrointestinal	295	3.8	288	3.8	358	0.9	1230	3.2	123	9.2	303	21.9
Mental health	3	0.0	3	0.0	19	0.0	21	0.1	0	0.0	0	0.0
Lifestyle	24	0.3	24	0.3	79	0.2	99	0.3	6	0.4	9	0.7

^#^Referrals on the same day were coded as post.

## Discussion

### Summary

This database study demonstrated that the prevalence of IBS in patients who consult, and are recorded by, their GP was low compared with the reported prevalence rates in the literature [2]. Most patients who had an IBS Read Code recorded did not have any gastrointestinal symptoms recorded prior to or after their index date. Likewise the majority of patients with AP or IBD did not have any symptoms recorded prior to or after their index date. Antispasmodics were the most commonly prescribed medications for patients with IBS. A significant proportion of patients were prescribed IBS NICE guideline recommended medications in the year prior to their IBS being Read Coded. A small proportion of patients with IBS were referred to GI specialists.

### Strengths and limitations

A major advantage of the SIR is the complete linkage between primary care, community care and secondary care datasets, providing a continuous electronic record for each patient in contact with health services in Salford. However, there were certain limitations in using these data for identifying patients with IBS in our study.

We were unable to validate the dataset. Ideally we would have created a cohort of IBS patients from SIR and validated the diagnosis by asking their GPs [10] but this was not possible since the SIR for research is anonymised at both patient and GP level. In addition, a number of computerised systems are available for capturing clinical consultation data in primary care and several different versions of the various operating systems are in use so there may be slight variations at computer operating system level that would have impeded our research.

There are also idiosyncrasies in the Read Code taxonomy, in particular the fact that codes for a lack of existence of a symptom can occur as leaf nodes describing that symptom. For example, the Read Code for “no abdominal pain” (1961.) is included under one of the Read Codes for abdominal pain (196..). However, it seems that GPs might be reluctant to document patients using such negated child node Read Codes as less than 0.1% were found in the data set we used.

Read Codes used to code a consultation are at the discretion of the individual clinician, which means that there can be considerable variation in their use to describe the same set of symptoms in practice (particularly for conditions not incentivised in the Quality Outcomes Framework (QOF)). However, research requires a disciplined approach to data entry and retrieval [11], so that inconsistency in coding potentially presents an important source of information bias.

### Recorded prevalence of IBS consultations

This study demonstrates that the recorded prevalence of IBS in patients who consult their GP is low. In the current study, we not only looked at the prevalence of IBS Read Codes in primary care, but the prevalence of AP, the main presenting feature for IBS. Even rates of AP were less than those reported for IBS in the literature. Reassuringly we found similar results using data extracted from Manchester Primary Care Organisation (data not shown) suggesting that the current findings are not an artefact of the data (Thompson DG, O’Brien S, Kennedy A, Rogers A, Whorwell P, Lovell K, *et al*.: Evaluating and Implementing Better Patient-Orientated Management of Chronic Gastrointestinal Disorders in Primary Care. *Programme Grants Appl Res* 2014, in review).

There are several possible explanations for the lower than expected prevalence of patients consulting with, and recorded as having, IBS.

Firstly, many of the studies estimating the prevalence of IBS have been self-report questionnaire surveys, and may have overestimated the proportion of patients who have been formally diagnosed with IBS, or who seek healthcare for their symptoms. Nevertheless the consultation prevalence for IBS was much lower than expected and the literature suggests that GPs see IBS as a significant problem [12]. Our findings reflect those in rheumatology where large discrepancies have been found in the consultation prevalence of knee pain when comparing primary care records with patient recall [13]. These discrepancies are as a result of 'telescoping’ by patients and under recording by GPs, in particular, if patients present with multiple problems or have previously consulted for the same condition [13]. Like knee pain, IBS does not have a definite diagnosis and symptoms fluctuate over time, thus primary care records may only reflect the consultation prevalence of IBS when it forms a major part of the consultation. Moreover, Jordan at el. [13] found that consultation rates increased when the text of the consultation was used in addition to Read Codes alone. Others have also discussed the benefit of narratives during the consultation rather than reducing the clinical encounter to a limited number of codes [14].

Secondly, IBS or GI symptoms, are not included in the Quality Outcomes Framework (QoF), a scheme that incentivises GP practices and rewards them according to how well they care for patients. Consequently, recording IBS is not likely to be a priority for GPs and therefore may be patchy.

Thirdly, GPs appear to be reluctant to code patients for IBS or lower GI symptoms. The IBS diagnosis in primary care appears to be different to, and less exclusive than existing diagnostic criteria [15]. The usefulness of diagnostic criteria is frequently debated in the literature [16-20] and they tend to be of little relevance within primary care, as few GPs are familiar with the criteria and they do not use them to make a diagnosis [17,21,22]. In our qualitative study to investigate how GPs defined, diagnosed and managed patients with IBS, we found that whilst most GPs were aware of the NICE guideline [4] for IBS, few used it to help them make a diagnosis of IBS and add a Read Code to the patient record [6]. Instead they described using an iterative process to exclude sinister symptoms and as a result perhaps not applying a Read Code to the patient record when they first consult.

Fourthly, recorded prevalence depends on the healthcare seeking behaviour of patients. Patients may not consult their GP for IBS for a number of reasons. They may only consult when their symptoms are severe and, consequently, they perhaps feel less able to cope. Indeed, data from our risk assessment study, where patients were recruited either via the consultation or through the use of Read Code searches (and had to have consulted for IBS symptoms in the last three months), suggest that the majority of patients had moderate or severe symptoms (Thompson DG, O’Brien S, Kennedy A, Rogers A, Whorwell P, Lovell K, *et al*.: Evaluating and Implementing Better Patient-Orientated Management of Chronic Gastrointestinal Disorders in Primary Care. *Programme Grants Appl Res* 2014, in review). Evidence suggests that symptom severity may have an influence on health care seeking but that it does explain the majority of the consultation behaviour [23]. Psychological and psychosocial factors have also been implicated in health care seeking behaviours for patients with IBS [24,25]. The present study suggests that a proportion of patients with IBS, AP and IBD were prescribed anti-depressants in the year before and the year after their index episode. However, we cannot infer whether these medications were prescribed for their gastrointestinal symptoms, for related anxiety and/or depression, or for unrelated anxiety and/or depression.

Furthermore, patients may not consult their GP for their IBS because their symptoms are under control through the use of medication or self-management. A relatively large proportion of patients in the current study had no GI symptoms in the year prior to their index episode of IBS but had been prescribed IBS medications, as recommended in the NICE guideline [4]. However, we are unable to say whether these patients had ever been recorded as having IBS prior to the year before their index episode, and whether they had received a repeat prescription from their GP for their symptoms.

Finally, patients may feel there is little that primary care can offer, and therefore learn to live with their symptoms. Stenner et. al. [26] reported that patients felt that doctors were unsympathetic and ignorant about IBS, and often considered IBS 'all being in the mind’ of the patient. Others felt GPs were responsible for the worsening of their condition as a result of their ignorance of IBS or 'through the iatrogenic effects of treatment’ [26]. Farndale et al. [27] found that IBS patients report alienation from health services for similar reasons. In addition, moderate to high levels of perceived stigma are significantly greater in IBS patients (27%) compared to IBD patients (8%), with the largest difference being for health care providers [28]. It is, therefore, perhaps unsurprising that a proportion of those suffering from the symptoms of IBS do not consult their GP and decide to self-medicate and/or seek alternative therapies. Although alternative therapies are not recommended within the NICE guidance [4] our qualitative study showed that GPs did not discourage their use [6].

### IBS and GI symptoms

Our study found that the majority of patients who were coded as having IBS did not have GI symptoms recorded in the year prior to or the year after their index episode. NICE recommends assessment for IBS in patients having any of the following for at least 6 months: abdominal pain or discomfort, bloating or change in bowel habit. Therefore one might expect evidence of this in the recording of symptom codes in the year prior to or after the index episode of IBS. However, this was not the case, despite using symptom codes in order to be as comprehensive as possible and by including other related symptoms, such as nausea and vomiting, tenesmus and faeces/motions. Interestingly though, IBS medications as recommended by NICE were often prescribed in those without recorded symptoms in the year prior to their IBS. GPs in the qualitative study also described making a diagnosis based on the patient response to a trial of medication [6]. This suggests that the use of medication codes may be an alternative approach to identifying patients via symptom or diagnostic Read Codes. GPs must enter a medication on their clinical system in order for a prescription to be issued. However this approach may include patients with other diagnoses as some IBS medications (e.g. laxatives) may also be prescribed for other conditions.

Findings from our qualitative study suggest GPs did not describe difficulties in managing patients with IBS [6]. This is also evident in the current study where a relatively small proportion of patients with IBS had a referral to a GI specialist, compared to those patients with a diagnosis of IBD.

### Similarities with other medically unexplained symptoms

Similarities can be seen with other medically unexplained symptoms such as fibromyalgia and chronic fatigue syndrome. For example, Rohrbeck et al. [29] found the recorded annual prevalence of fibromyalgia in primary care to be 8 per 10 000 which is much lower than the estimated general population prevalence of 2%. This implies that the label of fibromyalgia is rarely used within general practice [29]. They also found that fibromyalgia patients are similar to those with overlapping functional syndromes or medically unexplained symptoms [29]. Similarly, in his review of medically unexplained symptoms in primary care, Burton [30] reported that many patients with IBS met the criteria for fibromyalgia and chronic pelvic pain. This overlap with other medically unexplained symptoms, which often appear to share similar psychosocial characteristics, creates further diagnostic complexities.

## Conclusions

Our findings suggest that the use of symptom /and or diagnostic Read Codes to identify patients with IBS in primary care is questionable and likely to lead to large underestimates of both the community incidence and prevalence. The discrepancies between the self-reported prevalence rates in the literature and those for consultations within the primary care record, suggest that there may be conflicting priorities between patients and health care professionals, and that database studies, are useful in only identifying the 'tip of the iceberg’.

### NIHR disclaimer

This paper presents independent research commissioned by the National Institute for Health Research (NIHR) under its Programme Grants for Applied Research funding scheme (RP-PG-0407-10136). The views expressed in this paper are those of the authors and not necessarily those of the NHS, NIHR or the Department of Health.

### Ethics approval

Ethical permission for this study was granted by the North West e-Health Board (Reference 177), and the individual healthcare organisations.

## Appendix

Tables 7, 8 and 9.

**Table 7:** Symptoms/diagnoses were defined as any of the following

**Symptom/diagnoses**	**Read codes**	**Rubric**
Irritable bowel syndrome	14CF.	History of IBS
	J521.	IBS
Abdominal pain	196..	Type of gastrointestinal tract pain
	197..	Site of gastrointestinal tract pain
	R090.	[D] Abdominal pain
Bloating	19A..	Abdominal distension symptom
	19B..	Flatulence/wind
	R0734	[D] Bloating
Constipation symptom	19C..	Constipation
Functional constipation	J520.	Constipation - functional
Change in bowel habit	19EA.	Change in bowel habit
	R078.	[D] Change in bowel habit
Diarrhoea	19F..	Diarrhoea symptoms
	19G..	Diarrhoea and vomiting
Functional diarrhoea	J525.	Functional diarrhoea
	J43z.	Chronic diarrhoea
	J4z..	Presumed noninfectious diarrhoea
Nausea	198..	Nausea
Vomiting	199..	Vomiting
Diarrhoea and vomiting	19G..	Diarrhoea and vomiting
Tenesmus	19D..	Tenesmus symptom
Faeces/motions symptoms	19E..	Faeces/motions -symptoms
GI infection	A0…	Intestinal infectious diseases
Inflammatory bowel disease	J40..	Crohn’s disease
	J41..	Ulcerative colitis or proctitis

**Table 8:** **Medications were defined on the basis of the NICE guidelines**[4]**and were coded as follows**

**NICE medications**	**Read codes**	**Rubric**
Bulk-forming laxatives	ab2..	Isphagula husk
	ab3..	Methylcellulose
	ab4..	Sterculia
Stimulant laxatives	ac5..	Docusate sodium
	ac7..	Senna
	ac8..	Sodium picosulphate
	af1..	Rectal laxatives (Glycerol, biascodyl)
	ac1..	Biascodyl
Faecal softeners	ad1..	Liquid paraffin
Osmotic laxatives	ae4..	Polyethylene glycols
	a12..	Magnesium salts – antacid
	ae2..	Magnesium hydroxide
	ae3..	Magnesium sulphate
	ae7..	Sodium phosphate
Antimotility agents	a81..	Codeine phosphate
	a82..	Diphenoxylate hydrochloride
	a83..	Loperamide: single drug
	a85..	Loperamide: compound preparation
	a842.	Kaolin and morphine mixture
Antispasmodics	a41..	Atrophine sulphate
	a45..	Dicycloverine hydrochloride
	a47..	Hyoscine butylbromide
	a4c..	Propantheline bromide
	a4d..	Alverine citrate
	a4e..	Mebeverine hydrochloride
	a4f..	Peppermint oil
Antidepressants		
Tricyclics and related antidepressants	d71..	Amitripyline
	d91..	Triptafen
	d73..	Clomipramine hydrochloride
	d75..	Dosulephin hydrochloride
	d76..	Doxepin
	d77..	Imipramine hydrochloride
	d79..	Lofepramine
	d7c..	Nortriptyline
	d7f..	Trimipramine
	d7b..	Mianserin hydrochloride
	d7e..	Trazodone hydrochloride
	da9..	Citalopram
Selective serotonin re-uptake inhibitors (SSRIs)		
	daC..	Escitalopram
Monoamine oxidase inhibitors (MAOIs)	da4..	Fluoxetine hydrochloride
Reversible MAOIs	da3..	Fluvoxamine maleate
	da6..	Paroxetine hydrochloride
	da5..	Sertraline hydrochloride
Other antidepressants	d81..	Phenelzine
	d83..	Isocarboxazid
	d84..	Tranylcypromine
	d85..	Moclobemide
	gde..	Duloxetine
	da1..	Flupentixol
	daB..	Mirtazapine
	daA..	Reboxetine
	da2..	Tryptophan

**Table 9:** Referrals to specialists or for further for investigation of gastrointestinal symptoms were defined as any of the following

**Referral**	**Read codes**	**Rubric**
Specialist referral	8h48.	Gastroenterological referral
	8h5J.	Referral to colorectal surgeon
	8H5K.	Referral to upper gastrointestinal surgeon
	8HL8.	Gastroenterology DV done
	8HM8.	Listed for gasterenterol admis
	8Hn4.	Fast track referral for suspected colorectal cancer
	8Hn9.	Fast track referral for suspected upper GI cancer
	8HS..	Refer for gastroscopy
	8HS0.	Refer for sigmoidoscopy
	8HU1.	Referral for colonoscopy
	8HU2.	Referral for sigmoidoscopy
	8HVc.	Private referral to colorectal surgeon
	8HVN.	Private referral to gastroenterologist

## Competing interests

The authors declare that they have no competing interests.

## Authors’ contributions

EFH participated in the study design, supervised the data analysis, and led writing the paper. LG participated in data analysis and contributed to writing the paper. SJO’B participated in the study design and contributed to writing the paper. DGT participated in study design and contributed writing the paper. CCG contributed to writing the paper and led on the qualitative study. All authors read and approved the final manuscript.

## Pre-publication history

The pre-publication history for this paper can be accessed here:

http://www.biomedcentral.com/1471-2296/14/183/prepub
